# Commentary on the clinical management of metabolic syndrome: why a healthy lifestyle is important

**DOI:** 10.1186/1741-7015-10-139

**Published:** 2012-11-14

**Authors:** Michel de Lorgeril

**Affiliations:** 1 Laboratoire Cœur et Nutrition, TIMC-IMAG CNRS 5525, Université Joseph Fourier, Faculté de Médecine, 38054 La Tronche, Grenoble, France

**Keywords:** metabolic syndrome, meta-analysis, lifestyle, triglycerides, obesity, overweight, insulin resistance, diabetes, hypertension, high-density lipoprotein

## Abstract

Metabolic syndrome (MS) is associated with an increased risk of type 2 diabetes mellitus and cardiovascular diseases. There is no recognized method to manage MS. Many physicians treat the individual characteristics of MS (high blood pressure, high triglycerides, and so on) instead of the syndrome as a whole, placing particular emphasis on those components that are easily amenable to drug treatment. However, regular physical exercise and a healthy diet have been demonstrated to improve the health of a number of populations, but few studies have assessed their effects in patients with MS. A meta-analysis by Yamaoka and Tango in *BMC Medicine *found that a lifestyle change program (dietary counseling and encouragement to exercise) resulted in improvements in components of MS and in reducing the proportion of patients with MS. The effects may not be impressive in absolute terms, but the data should be interpreted with the heterogeneity of the included studies in mind. Because of the many adverse side effects of the drugs used to correct individual aspects of MS, this meta-analysis provides strong evidence that lifestyle changes must be the first-line approach to manage MS.

See related article http://www.biomedcentral.com/1741-7015/10/138/abstract

## Commentary

Metabolic syndrome (MS), defined as a cluster of biological and physiological characteristics, is associated with an increased risk of type 2 diabetes mellitus, cardiovascular disease (CVD), and all-cause mortality [1,2].

Several definitions of MS have been given, depending on the respective fields of specialty of the experts (cardiology, diabetes, obesity, and so on). According to the American Heart Association [1], MS is diagnosed when any three of the following criteria are associated: waist circumference >102 cm in men and >88 cm in women; triglycerides >150 mg/dl; high-density lipoprotein (HDL) cholesterol <40 mg/dl in men and <50 mg/dl in women; systolic blood pressure >130 mmHg, diastolic blood pressure >85 mmHg, or current use of antihypertensive medication; and fasting glucose >100 mg/dl or current drug treatment for elevated blood glucose.

The clinical management of MS is difficult because there is no recognized method to prevent or improve the whole syndrome, the background of which is essentially insulin resistance [3]. Therefore, many physicians, depending on their own medical subspecialty, treat one particular characteristic of MS (high blood pressure, high blood glucose, and so on) instead of the syndrome as a whole. However, one of the characteristics of MS is generally more degraded than the others in most patients (overweight, high triglycerides, and so on), and it is this one that patients usually wish to have corrected.

Thus, most physicians treat each component of MS separately, laying particular emphasis on those components that are easily amenable to drug treatment. In fact, it is easier to prescribe a drug to lower blood pressure, blood glucose or triglycerides rather than initiating a long-term strategy to change people's lifestyle (exercise more, eat better) in the hope that they will ultimately lose weight and tend to have lower blood pressure, blood glucose and triglycerides.

This is a critical issue because it is still not clear whether type 2 diabetes and CVD ultimately result from a deleterious lifestyle or from the specific abnormalities that characterize MS. In other words: is it the lack of physical exercise that is harmful, or a low HDL level?

Recent data regarding the use of drugs to raise HDL levels suggest that a marked increase in HDL does not result in significant health benefits, and that it may be more important to correct the lifestyle; that is, more skeletal muscle activity associated with more physical exercise rather than artificially increasing HDL using a drug [4-6].

In addition, there is still controversy regarding the best diet to reduce the risk of type 2 diabetes and to prevent or improve MS [7-12].

As a consequence, many physicians think that the first priority to protect their patients is to help them stop smoking and lower their blood cholesterol (usually with a statin), hoping that this approach will reduce the risk of CVD complications. However, both smoking cessation [13,14] and use of statins - causing a decrease in physical activity because of their toxicity on skeletal muscle [15,16]**- **can lead to weight gain, deterioration of some MS characteristics and increase the risk of new-onset type 2 diabetes [17,18].

It is only on reconsideration that some physicians may try to improve their patient's diet and encourage them to exercise more. At that time, as smoking cessation and lower cholesterol levels due to use of a statin may have considerably improved their risk factor status, patients might be unwilling to further change their lifestyle. Also, many of them may be reluctant to exercise because of the toxic effects of statins on their muscles. As a consequence, proactive physicians prescribe drugs to lower blood pressure, blood glucose and triglycerides to further improve the risk factor status of their patients, and many patients inevitably develop the many adverse effects of these drugs [19-22], in particular increased insulin resistance. This is definitely a vicious circle, and unfortunately a realistic description of what happens in daily medical practice.

And yet, regular physical exercise and a healthy diet have been unequivocally demonstrated to improve the health of a number of populations [23-27]. However, few studies have assessed their effects in individuals with MS [28-32].

In this context, the systematic review and meta-analysis performed by Yamaoka and Tango, examining the effects of lifestyle changes on MS, is welcome [33]. They have identified eight randomized controlled trials and included them in a meta-analysis. They found that a lifestyle change program (essentially dietary counseling and encouragement to exercise more) resulted in significant improvements in most components of MS and in reducing the proportion of patients with MS in comparison to conventional approaches [33].

The effects may not be impressive in absolute terms, but the data should be interpreted with the heterogeneity of the included studies in mind. In fact, variability is quite wide in terms of study duration and the types of lifestyle changes. For instance, some investigators used either the DASH ('Dietary Approaches to Stop Hypertension') or the Mediterranean diet, whereas others used low-fat diets.

The studied populations also are a source of heterogeneity. Some studies were conducted in Europe, either Northern Europe (UK and Finland) or Southern Europe (Italy and Spain), and others in the USA and in Iran. In fact, it is likely that the traditional lifestyles in these various parts of the world and the expected responses of the studied populations to any intervention will be different. Finally, there was no attempt in this meta-analysis to adjust for the drugs used in these highly diverse populations. As discussed above, drugs remain the preferred approach of many physicians in most European countries and in the USA to improve or suppress the abnormalities associated with MS. This factor may have been a major confounder in some of the included trials.

Despite these limitations, and also because of the many adverse side effects of the drugs used to correct some aspects of MS, as the authors conclude, this meta-analysis provides strong evidence that long-term lifestyle changes must be the first-line approach to reduce the prevalence of MS and of its complications.

## Competing interests

The author declares that he has no competing interests.

## Author information

MdeL is a cardiologist and nutritionist, and full-time researcher at the School of Medicine of the University of Grenoble (France) and at the French National Centre for Scientific Research (CNRS) in the Life Science Department.

## Pre-publication history

The pre-publication history for this paper can be accessed here:

http://www.biomedcentral.com/1741-7015/10/139/prepub
